# Case Report: autosomal dominant distal motor neuropathy as a new phenotype of *KIF21A*-related disorders

**DOI:** 10.3389/fgene.2025.1699834

**Published:** 2025-11-06

**Authors:** Dmitrii Subbotin, Eugene Tatarskiy, Anna Kuchina, Tatiana Cherevatova, Tatiana Krylova, Oksana Ryzhkova, Mikhail Skoblov, Aysylu Murtazina

**Affiliations:** Research Centre for Medical Genetics, Moscow, Russia

**Keywords:** peripheral neuropathy, KIF21A, expanding phenotype, hereditary distal motor neuropathy, dHMN

## Abstract

Heterozygous missense variants in the *KIF21A* gene are best known to cause congenital fibrosis of the extraocular muscles. A recent report by Borja et al., 2025 suggested that the *KIF21A* gene may also be associated with syndromic phenotype, including peripheral neuropathy, brain malformations, and strabismus. We report the second case of early-onset distal motor neuropathy associated with the *KIF21A* gene. The proband was a 6-year-old female patient who had normal brain MRI, while neurophysiological examination and lower limb muscle MRI both suggested peripheral neuropathy. Quad whole-genome sequencing of the proband, her healthy sibling, and parents identified a *de novo* missense variant, c.1991T>C, p. (Leu664Pro), in the *KIF21A* gene and two compound-heterozygous missense variants, c.274C>T, p. (Pro92Ser) and c.512A>G, p. (Asn171Ser), in the *OXA1L* gene. Since the clinical features were not fully consistent with the known phenotypes associated with *KIF21A* or *OXA1L*-related disorders, initial genetic analysis prioritized *OXA1L*. However, functional studies, including exploratory Western blot analysis and high-resolution respirometry failed to support the pathogenicity of the identified variants. Following the publication of a similar *KIF21A*-associated case, the c.1991T>C, p. (Leu664Pro) variant was re-evaluated and re-classified as likely pathogenic. Our case supports expansion of the *KIF21A*-related phenotype to include distal motor neuropathy without brain malformations, in addition to multiple reports of other *KIF21*-associated syndromic phenotypes. This finding suggests that the *KIF21A* gene should be considered in the differential diagnosis for patients presenting with childhood-onset distal motor neuropathies.

## 1 Introduction

Distal hereditary motor neuropathy (dHMN) is a clinically and genetically heterogeneous group of disorders, characterized by selective impairment of lower motor neurons and/or motor nerve fibers (Harding and Thomas, 1980). These diseases are characterized by slowly progressive symmetrical weakness and atrophy of the distal muscles and absence or minor sensory abnormalities, as assessed both clinically and electrophysiologically. To date, approximately 30 genes are associated with dHMN (Tazir and Nouioua, 2024). The significant phenotypic overlap between dHMN and other neuropathies and motor neuron disorders, often accompanied by a variety of additional features, poses considerable challenges for differential diagnosis. This complexity underscores the need to expand the known genetic spectrum associated with this group of disorders.

The *KIF21A* gene encodes an anterograde kinesin motor protein, which belongs to a family of kinesin motor proteins (Marszalek et al., 1999). These proteins play a significant role in transporting cellular components within cells. In addition, KIF21A directly modulates microtubule dynamics by accumulating at microtubule plus ends and acting as a cortical inhibitor of microtubule growth, thereby reducing microtubule growth rates (van der Vaart et al., 2013). Dysregulation of axonal transport with secondary axonal degeneration is a known mechanism in numerous neurodegenerative diseases and neuronopathies, including amyotrophic lateral sclerosis, hereditary motor and sensory neuropathies, and dHMN/distal spinal muscular atrophy (Berth and Lloyd, 2023). These pathways suggest a wider phenotypic spectrum for *KIF21A*-related disease.

The *KIF21A* gene is associated with several clinical phenotypes. The most frequent is congenital fibrosis of the extraocular muscles (CFEOM; OMIM #135700) caused by heterozygous missense variants through a gain-of-function mechanism, classically presenting as isolated, non-progressive ophthalmoplegia with or without ptosis (Yamada et al., 2003; Cheng et al., 2014). In addition to isolated CFEOM, syndromic *KIF21A* conditions were reported, in which CFEOM features co-occurred with extra-ocular symptoms such as cerebellar vermis hypoplasia and ataxia (Di Fabio et al., 2013). One report described a patient with a syndromic phenotype that included intellectual disability, peripheral neuropathy, and brain abnormalities (Soliani et al., 2021). By contrast, biallelic loss-of-function *KIF21A* variants cause a severe fetal akinesia and distal arthrogryposis phenotype, often with brain malformations (Falb et al., 2023; Bhola et al., 2024). Recently, a case of a *KIF21A*-associated syndromic phenotype characterized by progressive peripheral neuropathy, hypoplasia of the splenium of the corpus callosum, and strabismus has been described described (Borja et al., 2025).

The *OXA1L* gene encodes the human inner mitochondrial membrane insertase that mediates the insertion of mtDNA-encoded respiratory chain subunits and supports the assembly of the oxidative phosphorylation (OXPHOS) complexes by coupling to the mitoribosome (Stiburek et al., 2007; Itoh et al., 2021). Biallelic variants in the *OXA1L* gene have been reported to cause severe early-onset mitochondrial encephalopathy and mitochondrial myopathy with combined OXPHOS deficiencies in patient-derived cells and model systems (Thompson et al., 2018; Zhan et al., 2025). In the previous *OXA1L* cases in the patient cells exhibited impaired oxidative phosphorylation complex subunits assembly and depressed cellular respiration, which has been demonstrated by abnormal fibroblast respirometry (Thompson et al., 2018).

Here we reported the second case p. (Leu664Pro) *KIF21A* gene variant of a proband with early childhood-onset distal motor neuropathy who underwent quad whole genome sequencing (WGS) along with her family members.

## 2 Subjects and methods

The clinical presentation of the patient was thoroughly evaluated. The diagnostic workup included nerve conduction studies and needle electromyography of clinically affected muscles, the magnetic resonance imaging (MRI) of brain and lower limbs muscles. The quad WGS was performed for the proband, her sibling and parents.

For functional analysis, primary skin fibroblast cultures were derived from inner forearm skin biopsies of the proband, his parents and ten healthy volunteers and deposited in the Moscow Branch of the Biobank « Russian collection of biological samples of hereditary diseases».

Exploratory Western blot analysis was performed on patient-derived fibroblasts, comparing them to parental and unrelated controls (n = 1). Protein lysates were probed with an anti-OXA1L antibody (Proteintech, Cat# 21055-1-AP, RRID:AB_10695769) and normalized to β-Actin (Sigma-Aldrich, Cat# A1978, RRID:AB_476692).

3D structural analysis of the KIF21A protein was performed for comparing the wild-type and the p.Leu644Pro mutant protein structures. Protein structures were predicted using the OmegaFold and ColabFold, which utilizes the AlphaFold2 architecture with MMseqs2 for multiple sequence alignment (Mirdita et al., 2022; Wu et al., 2022). For each tool, both the wild-type and mutant sequences were submitted, and five models were generated per run to assess prediction variation. The resulting models were visualized, aligned, and analyzed using PyMOL Molecular Graphics System (Version 2.5.8 Schrödinger, LLC). Structural alignment was performed on a single, conserved alpha-helix to facilitate the comparison of conformational changes induced by the mutation.

High-resolution respirometry was performed using the skin fibroblasts from the patient and controls (n = 10) according to the protocol described earlier (Krylova et al., 2025). Primary skin fibroblast cultures were derived from inner forearm skin biopsies using standard fibroblast cell culture techniques. The primary fibroblast cultures were grown in “Amniokar” proliferative medium (PanEco-Ltd, Moscow, Russia). Further subculturing of the fibroblasts was performed in Dulbecco Modified Eagle Medium growth medium supplemented with 10% FBS (PanEco-Ltd). The skin fibroblasts from the patient and controls (n = 10) were harvested using 0.25% trypsin and centrifuged at 1500 rpm for 5 min. The pellet (1.5−3 × 106 cells/mL) was resuspended in a pre-warmed (at 37 °C) respiration medium MIR05 (100 mM sucrose, 60 mM potassium lactobionate, 0.5 mM EGTA, 3mM MgCl2x 6H2O, 20 mM taurine, 10 mM KH2PO4, 20 mM HEPES, and 1 mg/mL BSA, pH = 7.1). The analysis of oxygen consumption was performed with the Oxygraph-2k (Oroboros Corp., Innsbruck, Austria) using DatLab 7.0 software. Experiments with intact cells were performed according to the protocol of Pesta and Gnaiger as described previously (Pesta and Gnaiger, 2012; Krylova et al., 2025). Flux Control Ratios (FCRs) were used to estimate bioenergetic defects: R/E, L/E, netR/E ((R-L)/E). R–ROUTINE–oxygen flux of living cells in MIR05 without exogenous substrates, L–LEAK–oxygen flux after 2.5 µM oligomycin addition, E–ETS–maximum oxygen flux by stepwise injection of 0.05 µM carbonyl-cyanide p- (trifluoromethoxy) phenylhydrazone (FCCP). Experiments were performed in duplicate (Pesta and Gnaiger, 2012; Krylova et al., 2025).

The patient’s parents provided written informed consent for anonymized data publication.

## 3 Case descriptions

The proband was a 6-year-old Russian girl who presented with severe gait disturbance and limited mobility of the interphalangeal joints of the hands. She was born to non-consanguineous Russian parents and had one healthy older brother. Family history was unremarkable, and she was the only affected family member. Her first symptom was delayed motor development, she began walking independently at 18 months, with a slow gait and frequent falls. Her cognitive function and speech development were within normal age-appropriate limits. Notably, her early medical history was significant for recurrent, forceful vomiting, with a variable frequency ranging from several times daily to 4–5 times per month without identifiable triggers during the first 2 years of life. Shortly after onset, she was hospitalized for evaluation, and no objective cause was identified. According to the parents, no further hospitalizations were required thereafter. The vomiting gradually decreased in frequency and resolved completely by the age of 5 years. Additional features included pes cavus and ankle contractures, noted at the age of 2 years. The parents also reported intermittent exotropia over the past year, with episodes occurring almost daily, lasting several seconds, and resolving spontaneously. The patient did not report associated diplopia or some discomfort. At age 5, during planned hospitalization, an electroencephalography showed no epileptiform activity. Also, during this period, she developed persistent dysarthria.

**FIGURE 1:**
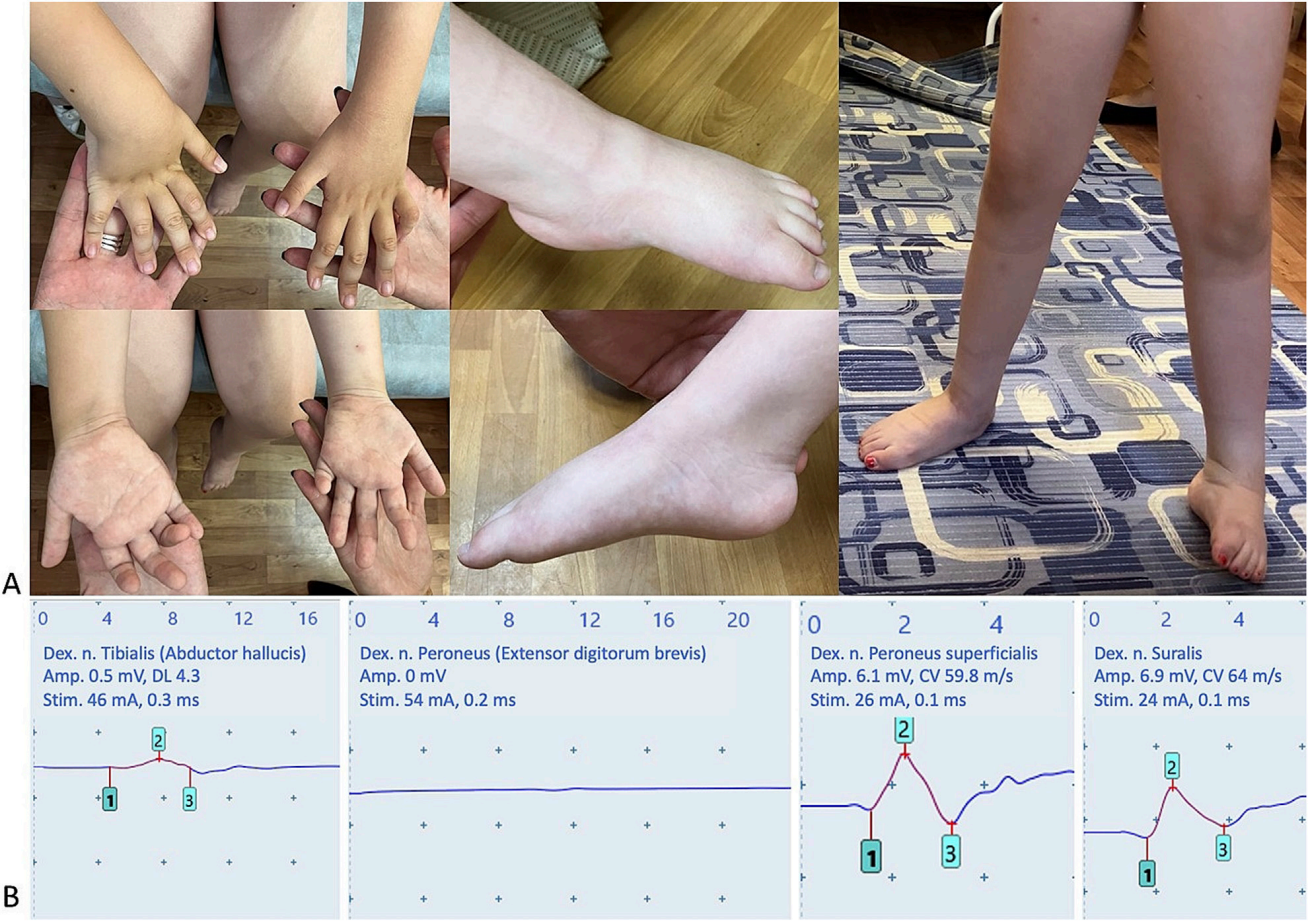
Clinical features and electrophysiological studies of the proband. **(A)** Distal muscle atrophy of the upper and lower limbs, ankles and hand interphalangeal joints contractures, valgus deformity of right foot and cavovarus deformity of left foot. **(B)** Nerve conduction study in lower limbs demonstrated normal sensory nerve action potentials in the peroneus superficialis and suralis nerves, and pronounced decreased compound muscle action potential amplitude in the tibial and peroneal nerves. Amp., amplitude; dex., dextra; DL, distal latency; Stim., stimulation parameters.

At age 6, clinical examination showed height 110 cm (−0.83 SD), weight 22.5 kg (+0.55 SD), and head circumference 51.5 cm (+0.2 SD). She had a normal body habitus with no dysmorphic features and no abnormalities in other organ systems. Neurologic examination revealed dysarthria without other bulbar abnormalities. Examination also revealed atrophy of the forearms, hands, the peroneal muscles, and the feet including the extensor digitorum brevis muscle (Figure 1A). Muscle strength was reduced to 3/5 on the Medical Research Council scale (MRC) in proximal muscles of both upper and lower limbs, 0/5 in intrinsic hand muscles, 0/5 in ankle dorsiflexors, and up to 2/5 in plantar flexors. Interestingly, neck flexor weakness was also noted. Contractures were observed at the ankles and interphalangeal joints of the hands. Deep tendon reflexes were reduced in the upper limbs and absent in the lower limbs. Both axial and appendicular tones were normal, no muscle hypertonia or hypotonia were noted. The patient’s sensory examination (touch, pain, vibration, proprioception senses) showed no abnormalities. Lumbar hyperlordosis, valgus deformity of right foot and cavovarus deformity of left foot were noted. The patient also demonstrated a positive Gowers’ sign and waddling gait with steppage. We observed several brief episodes of intermittent exotropia during the neurological exam, which resolved immediately upon voluntary fixation. No persistent deviation was noted between episodes.

Serum creatine kinase level was 380 U/L (reference 0–149 U/L). Nerve conduction studies showed motor axonal neuropathy in both upper and lower limbs (Figure 1B). The amplitude of the compound muscle action potential (CMAP) in the right median, ulnar and tibial nerves were significantly reduced (0.7mV, 0.9 mV and 0.5 mV, respectively), while conduction velocities in the median and ulnar nerves were within normal limits. CMAPs were absent in the peroneal nerves bilaterally. Sensory nerve action potentials in the upper and lower limbs were completely spared. Needle electromyography confirmed a clear neurogenic pattern, showing a decreased recruitment pattern with high-amplitude, long-duration motor unit potentials in the left tibialis anterior muscle.

**FIGURE 2:**
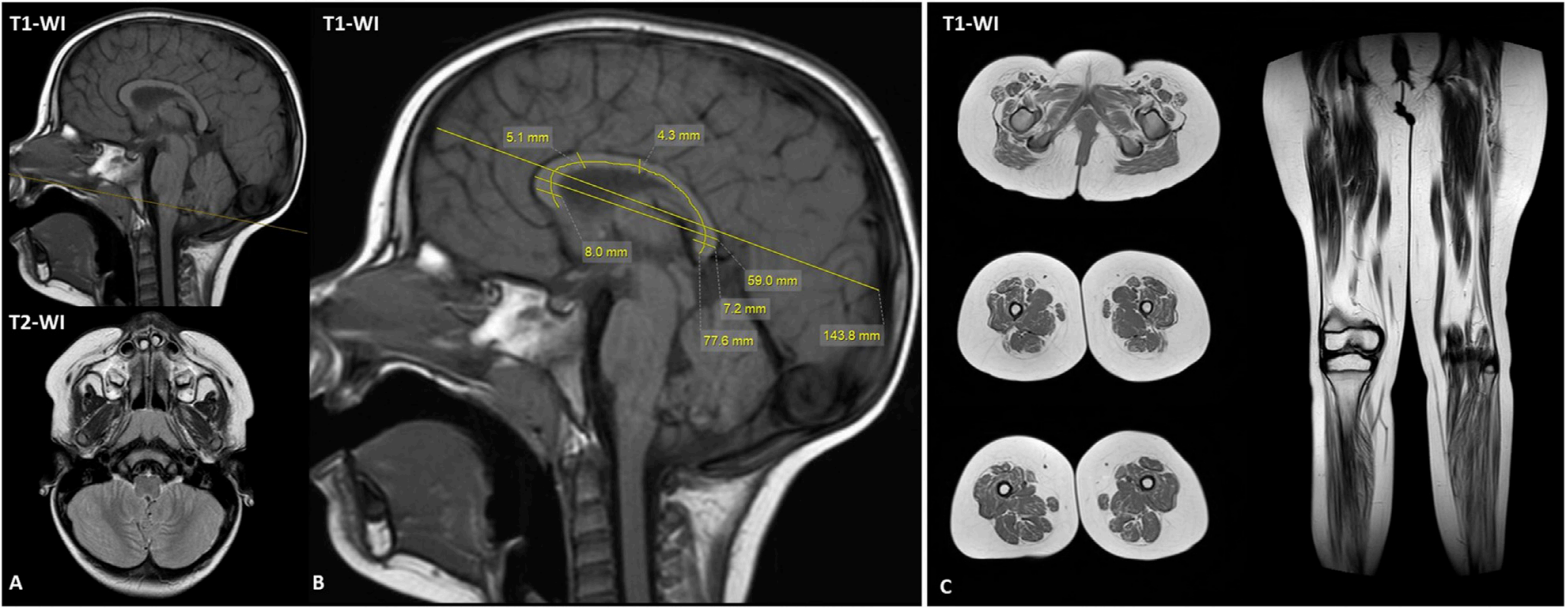
Magnetic resonance imaging of brain and lower limb muscles. **(A)** Brain MRI shows no structural brain abnormalities. **(B)** The brain MRI demonstrated normal range corpus callosum measurements for 3-year-old female proband. **(C)** The lower limb muscle MRI revealed diffuse fatty infiltration predominant in lower legs. T1-WI, T1 weighted image; T2-WI, T2 weighted image.

The brain MRI at the age of 3 years appeared unremarkable, without any structural brain anomalies (Figure 2). Corpus callosum morphology was carefully evaluated in the patient (Figure 2B). Measurements were compared with the age- and sex-specific centile tables followed by (Garel et al., 2011), and all fell within the normal range (between the 3rd and 97th centiles) for 3-year-old girls. Lower limb muscle MRI demonstrated diffuse fatty infiltration of lower leg and thigh muscles on T1-weighted images, indicative with a neurogenic pattern (Figure 2C). The adductor longus and adductor magnus were relatively spared. T2 STIR images showed hyperintensity in the posterior compartment of lower leg muscles.

Quad WGS identified the *de novo* missense variant c.1991T>C, p. (Leu664Pro) in the *KIF21A* gene (NM_001173464.2) and two compound-heterozygous missense variants, c.274C>T, p. (Pro92Ser) and c.512A>G, p. (Asn171Ser), in the *OXA1L* gene (NM_005015.5). According to gnomAD (v3.1.2), the variants c.1991T>C in the *KIF21A* and c.274C>T in the *OXA1L* were absent in general population database and the variant c.512A>G in the *OXA1L* occurs in East Asian, Admixes American, and European populations at a frequency of 0.000046, with no homozygotes recorded. Initially, all variants were classified as variants of uncertain significance under ACMG criteria (PM2) (Richards et al., 2015).

During our initial NGS (Next-Generation Sequence) data analysis, the ClinVar database contained only two submissions for the for the *KIF21A* variant c.1991T>C p. (Leu664Pro), both lacking phenotype annotation (RCV000497582.2; RCV003159607.2). At that time, because the *KIF21A*-associated CFEOM phenotype in OMIM did not match the proband’s clinical presentation, we first prioritized functional assessment of the *OXA1L* gene variants.

The c.512A>G variant in the *OXA1L* gene is located in the same transmembrane domain as previously reported pathogenic variants associated with mitochondrial encephalomyopathy and myopathy (Thompson et al., 2018; Zhan et al., 2025). The second c.274C>T variant is outside that local domain, is absent from gnomAD v3.1.2, and occurs in trans with c.512A>G. The other variant was outside the described domain, but was not found in the gnomAD (v3.1.2) database and was in a compound heterozygous state. Despite being classified the variants in the *OXA1L* gene as non-deleterious by the *in silico* predictor AlphaMissense (deleteriousness scores: p.Pro92Tyr = 0.210; p.Asn171Ser = 0.201), we decided to functionally characterize the two identified variants. Given the high endogenous expression of OXA1L in fibroblasts, we performed exploratory Western blot analysis on patient-derived fibroblasts, comparing them to parental and unrelated controls (n = 1). This analysis revealed no detectable differences in OXA1L protein abundance or electrophoretic mobility (Supplementary Figure S1A).

High-resolution respirometry was performed using intact fibroblast cell lines from the patient and controls (n = 10). No significant differences in Flux Control Ratios were found between the patient and control group (Supplementary Figure S1B). Abnormal fibroblast respirometry would be expected if the *OXA1L* variants were truly pathogenic. This has been demonstrated in previous *OXA1L* cases, where patient cells exhibited impaired oxidative phosphorylation complex subunits assembly and depressed respiration (Thompson et al., 2018).

According to the results of Western blot analysis and high-resolution respirometry the *OXA1L* variants were considered unlikely to be causative. So, the *OXA1L* gene variants c.274C>T, p. (Pro92Ser) and c.512A>G, p. (Asn171Ser) were reclassified as likely benign based on the functional studies and ACMG criteria (PM2, BS3, BP7) (Richards et al., 2015).

Although initial evaluations were non-diagnostic, the *de novo KIF21A* variant was re-evaluated following its updated annotation in ClinVar with the proband’s phenotype, prompted by the case report by Borja et al., 2025, which describes a patient with a similar phenotype (Borja et al., 2025). The concordant phenotype, *de novo* status, and high *in silico* scores (AlphaMissense 0.9997; CADD 28.9) allowed reclassification of c.1991T>C, p. (Leu664Pro) as likely pathogenic, according to ACMG criteria (PS2, PM2, PP3) (Richards et al., 2015).

**FIGURE 3:**
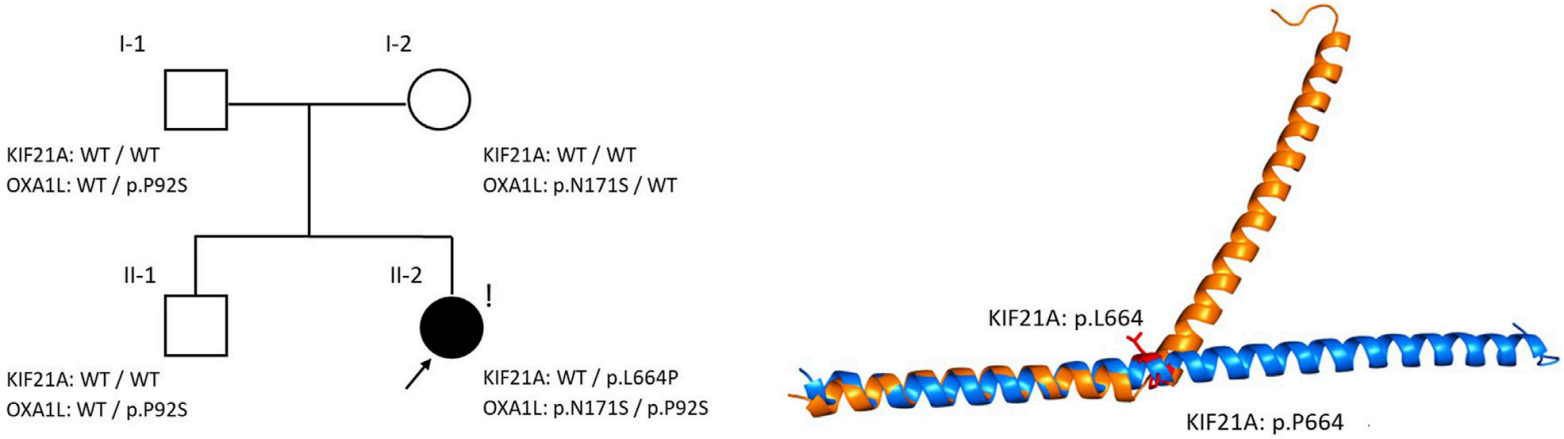
The pedigree of the proband and structural alignment of the KIF21A α-helix with WT (blue) and with p. (Leu664Pro) (orange).

Previous *in vitro* studies demonstrated that the c.1991T>C p. (Leu664Pro) variant alters the structure of the KIF21A protein and disrupts its interaction with TUBB3, a microtubule component critical for peripheral axon growth and regeneration (Tischfield et al., 2010; Borja et al., 2025). The predictive tool AlphaFold2, while excellent for wild-type structures, is often ill-suited for predicting conformational changes induced by missense variants (Agarwal and McShan, 2024). To overcome these limitations and better investigate the structural impact, we used OmegaFold, an alternative predictive tool that operates independently of multiple sequence alignments (Wu et al., 2022). The OmegaFold prediction successfully revealed a distinct helix break at the proline 664 position (Figure 3). This finding suggests that the structural disruption caused by the p. (Leu664Pro) variant is severe, providing a plausible explanation for the profound disruption in TUBB3 binding observed experimentally.

Due to the lack of specific therapy, the patient was given only general recommendations for physiotherapy, and dynamic evaluations by a neurologist, an ophthalmologist, and an orthopedic surgeon.

## 4 Discussion

We described a 6-year-old female patient with a predominant phenotype of distal motor neuropathy who carries the heterozygous *de novo* missense variant c.1991T>C, p. (Leu664Pro) in the *KIF21A* gene. At the time of initial analysis of the NGS data, no publications had clearly linked the *KIF21A* gene to an isolated distal motor neuropathy, and the c.1991T>C, p. (Leu664Pro) variant in *KIF21A* was therefore not prioritized. By contrast, OMIM lists no established phenotype for the *OXA1L* gene, however, biallelic *OXA1L* gene variants have been associated with mitochondrial disease (Thompson et al., 2018; Zhan et al., 2025). Consequently, we first focused on evaluating the detected biallelic variants in the *OXA1L* gene. However, the absence of aberrant protein expression and normal respirometry results suggested that the *OXA1L* variants were unlikely to be pathogenic. The subsequent publication by Borja et al. (2025), who described the patient with a similar phenotype and the same variant, prompted the re-evaluation of our case and resolution of the diagnostic challenge (Borja et al., 2025).

The phenotypic spectrum of the *KIF21A*-related disorders is broad and includes CFEOM, and several reports linking them to inherited distal arthrogryposis (Engle et al., 1997; Falb et al., 2023). Some clinical-phenotypic correlations have been established. Specifically, heterozygous missense variants are most frequently associated with CFEOM, a congenital, non-progressive ophthalmoplegia with or without ptosis and usually without central nervous system malformations (Yamada et al., 2003). CFEOM results from a malformation of cranial motor nerves and their neurons (Demer et al., 2005; Cheng et al., 2014). The oculomotor nerve is consistently the most markedly hypoplastic cranial nerve, though hypoplasia of the abducens nerve is also observed. Although extraocular manifestations are less common, several patients with typical congenital bilateral ptosis/ophthalmoparesis have shown cerebellar vermis atrophy and adult-onset cerebellar signs, without peripheral neuropathy (Di Fabio et al., 2013). Biallelic loss-of-function *KIF21A* variants were associated with more severe phenotypes, as a fetal akinesia with multiple arthrogryposis, brain malformations, pulmonary hypoplasia, and facial dysmorphisms (Falb et al., 2023; Bhola et al., 2024). The isolated distal motor phenotype was not previously described in the literature. Notably, although the corpus callosum of our patient appeared similar to that in the case report of Borja et al., all measurements fell within age-appropriate normal and we also did not observe other structural brain abnormalities. Our proband was clinically diagnosed with distal motor neuropathy supported by the nerve conduction study, needle electromyography and lower limb muscle MRI findings. Notably, the proband’s medical history also included reports of forceful, unprovoked vomiting during the first years of life, as reported in the previous *KIF21A* p. (Leu664Pro) case. Interestingly, our patient’s intermittent exotropia contrasts with the persistent strabismus seen in (Borja et al., 2025). The phenotype of motor neuropathy clearly predominates in our proband (Table 1).

**TABLE 1:** Comparison of the proband phenotype in the current study and the previous reported case (Borja et al., 2025).

Features	Proband of Borja NA et al.	Current proband
Genetic variant	NM_001173464.2(KIF21A):c.1991T>C p. (Leu664Pro)	
Age at last exam (years)	7	6
Nervous system		
CFEOM	no	no
Strabismus	persistent exotropia	intermittent exotropia
Vocal cord dysfunction	yes	no
Dysphagia	yes	no
Dysartria	no	yes
Peripheral motor neuropathy	yes	yes
Peripheral sensory neuropathy	no	no
Development		
Hypotonia	yes	no
Motor delay	yes	yes
Speech delay	yes	no
Intellectual disability	yes	no
Other Features		
Hypertension	yes	no
Chronic respiratory disease	yes	no
GI dysmotility	yes	no
Frequent vomiting	yes	yes
Joint contractures	no	yes
Osteoporosis	yes	no
Nephrolithiasis	yes	no
Brain MRI Anomalies		
Abnormal corpus callosum	thin splenium	no
Abnormal brainstem	no	no

The patient’s diagnostic odyssey with comprehensive clinical and neurophysiological assessment, quad-WGS, and functional testing of alternative variants of uncertain significance, allowed us to establish the diagnosis and confirm the association of dHMN with the variant c.1991T>C, p. (Leu664Pro) in the *KIF21A* gene. This case expands the phenotypic spectrum of *KIF21A*-related disorders and provides additional evidence of the pathogenicity of this previously reported variant (Borja et al., 2025).

## 5 Conclusion

This study adds evidence that the *KIF21A* gene phenotypic spectrum includes distal motor neuropathy and suggests that it should be considered for genetic testing of peripheral neuropathies. Also provides functional data on two previously undescribed *OXA1L* variants excluding their associating with the patient’s disease. These findings highlight the phenotypic heterogeneity of *KIF21A*-related disorders and the need for its further investigation in larger cohorts.

## Data Availability

The data that support the findings of this study are available on request from the corresponding author. The data are not publicly available due to privacy or ethical restrictions.
