# Supplementary material for: Case Report: autosomal dominant distal motor neuropathy as a new phenotype of KIF21A-related disorders
Source: Front Genet. 2025 Nov 6;16:1699834. doi: 10.3389/fgene.2025.1699834 (PMC12631304; doi:10.3389/fgene.2025.1699834)
Supplement: Supplementary file 1 [file DataSheet1.pdf]

Supplementary Material

1 Supplementary Data

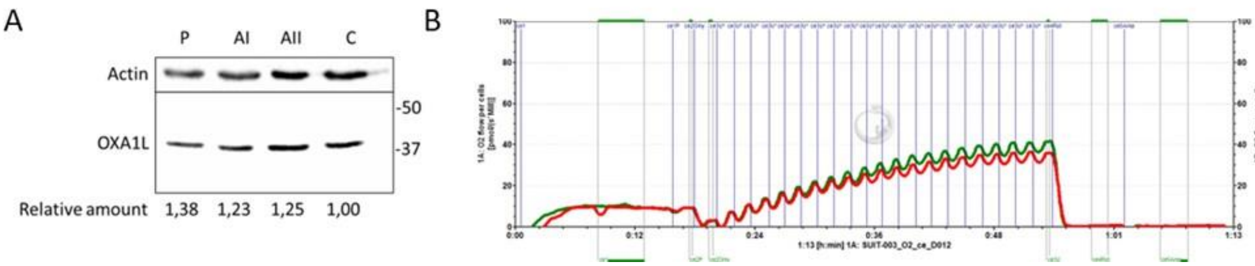

Supplementary Figure 1. Functional analysis of *OXA1L* variants. (A) Western blot analysis of OXA1L expression in fibroblasts from the proband, parents, and a control individual. Actin was used for normalization. (B) High-resolution respirometry performed using intact fibroblast cell lines from the patient (red line) and controls (green line) (n = 10). P, proband; AI and AII, parents; C, control individual.
